# Risk stratification for prostate cancer management: value of the Cambridge Prognostic Group classification for assessing treatment allocation

**DOI:** 10.1186/s12916-020-01588-9

**Published:** 2020-05-28

**Authors:** M. G. Parry, T. E. Cowling, A. Sujenthiran, J. Nossiter, B. Berry, P. Cathcart, A. Aggarwal, H. Payne, J. van der Meulen, N. W. Clarke, V. J. Gnanapragasam

**Affiliations:** 1 Department of Health Services Research and Policy, London School of Hygiene and Tropical Medicine, London, UK; 2 Clinical Effectiveness Unit, The Royal College of Surgeons of England, London, England; 3 Department of Urology, Guy’s and St Thomas’ NHS Foundation Trust, London, UK; 4 Department of Cancer Epidemiology, Population, and Global Health, King’s College London, London, UK; 5 Department of Radiotherapy, Guy’s and St Thomas’ NHS Foundation Trust, London, UK; 6 Department of Oncology, University College London Hospitals, London, UK; 7 Department of Urology, The Christie NHS Foundation Trust, Manchester, UK; 8 Department of Urology, Salford Royal NHS Foundation Trust, Salford, UK; 9 Academic Urology Group, Department of Surgery, University of Cambridge, Cambridge, UK; 10 Department of Urology, Cambridge University Hospitals NHS Foundation Trust, Cambridge, UK; 11 Cambridge Urology Translational Research and Clinical Trials Office, Cambridge Biomedical Campus, Cambridge, UK

**Keywords:** Prostate cancer, Non-metastatic disease, Risk stratification, Cambridge Prognostic Groups, CPG, Treatment selection

## Abstract

**Background:**

The five-tiered Cambridge Prognostic Group (CPG) classification is a better predictor of prostate cancer-specific mortality than the traditional three-tiered classification (low, intermediate, and high risk). We investigated radical treatment rates according to CPG in men diagnosed with non-metastatic prostate cancer in England between 2014 and 2017.

**Methods:**

Patients diagnosed with non-metastatic prostate cancer were identified from the National Prostate Cancer Audit database. Men were risk stratified according to the CPG classification. Risk ratios (RR) were estimated for undergoing radical treatment according to CPG and for receiving radiotherapy for those treated radically. Funnel plots were used to display variation in radical treatment rates across hospitals.

**Results:**

A total of 61,999 men were included with 10,963 (17.7%) in CPG1 (lowest risk group), 13,588 (21.9%) in CPG2, 9452 (15.2%) in CPG3, 12,831 (20.7%) in CPG4, and 15,165 (24.5%) in CPG5 (highest risk group). The proportion of men receiving radical treatment increased from 11.3% in CPG1 to 78.8% in CGP4, and 73.3% in CPG5. Men in CPG3 were more likely to receive radical treatment than men in CPG2 (66.3% versus 48.4%; adjusted RR 1.44; 95% CI 1.36–1.53; *P* < 0.001). Radically treated men in CPG3 were also more likely to receive radiotherapy than men in CPG2 (59.2% versus 43.9%; adjusted RR, 1.18; 95% CI 1.10–1.26). Although radical treatment rates were similar in CPG4 and CPG5 (78.8% versus 73.3%; adjusted RR 1.01; 95% CI 0.98–1.04), more men in CPG5 had radiotherapy than men in CPG4 (79.9% versus 59.1%, adjusted RR 1.26; 95% CI 1.12–1.40).

**Conclusions:**

The CPG classification distributes men in five risk groups that are about equal in size. It reveals differences in treatment practices in men with intermediate-risk disease (CPG2 and CPG3) and in men with high-risk disease (CPG4 and CPGP5) that are not visible when using the traditional three-tiered risk classification.

## Introduction

The Cambridge Prognostic Group (CPG) classification provides a five-tiered prostate cancer risk classification for non-metastatic prostate cancer. It has been shown to be a better predictor of prostate cancer deaths than traditional three-tiered classifications including those recommended by the UK National Institute for Health and Care Excellence (NICE) and the European Association of Urology (EAU) [1, 2].

The CPG classification was developed in an unscreened primary diagnostic cohort, encompassing all treatment types, making it truly a representative of a contemporary, real-world population [3]. A follow-up validation study confirmed that the five-tiered classification better predicted prostate cancer death across different ages and treatment groups than three-tiered classifications [4].

The core differences between the CPG system and the traditional three-tiered systems are the subdivision of intermediate-risk disease into CPG2 with favourable features (Gleason score 3 + 4 or PSA 10–20) and CPG3 with unfavourable features (Gleason score 3 + 4 and PSA 10–20, or Gleason score 4 + 3) as well as the subdivision of high risk into CPG4 (one high-risk feature of Gleason score 8, PSA > 20 or T3) and CPG5 (more than one high-risk feature of Gleason score 9–10 or T4) (Table 1) [3, 5]. With this finer degree of granularity, the CPG classification is the first reported classification system of prostate cancer risk that incorporates recommendations of the International Society of Urological Pathology on the grading of prostate cancer [6]. The American Urological Association [7] and the National Comprehensive Cancer Network [8] have both adopted subgroups for intermediate- and high-risk disease, but recent work has now shown that the CPG classification outperforms even these other approaches in head-to-head comparisons [2].

**Table 1:** Patient characteristics of men diagnosed with non-metastatic prostate cancer receiving radical treatment according to the Cambridge Prognostic Group (CPG) classification

CPG	Criteria	10-year risk of dying from prostate cancer without treatment* (%)	10-year risk of dying from prostate cancer with treatment* (%)
1	Gleason score 6 (grade group 1) *AND* PSA < 10 ng/ml *AND* stages T1–T2	4.2	1.2
2	Gleason score 3 + 4 = 7 (grade group 2) *OR* PSA 10–20 ng/ml *AND* stages T1–T2	4.7	2.3
3	Gleason score 3 + 4 = 7 (grade group 2) *AND* PSA 10–20 ng/ml *AND* stages T1–T2 *OR* Gleason score 4 + 3 = 7 (grade group 3) *AND* stages T1–T2	15.1	3.2
4	One of the following: Gleason score 8 (grade group 4) *OR* PSA > 20 ng/ml *OR* stage T3	N/A**	5.8
5	Any combination of Gleason score 8 (grade group 4), PSA > 20 ng/ml or stage T3 *OR* Gleason score 9–10 (grade group 5) *OR* stage T4	N/A**	13.7

*Mortality is taken from Gnanapragasam et al. [3]

*No reliable data is available for untreated men with CPG4 or CPG5

The National Prostate Cancer Audit (NPCA) has been collecting prospective data on every man newly diagnosed with prostate cancer in England and Wales since April 2014. So far, the Audit has only used the three-tiered classification system [9]. The aim of this study was to assess the disease presentation and treatment selection for men diagnosed with non-metastatic prostate cancer in England according to the CPG risk classification system.

## Methods

### Patient population

Our study used the NPCA database to identify 118,526 men diagnosed with non-metastatic prostate cancer between April 1, 2014, and March 31, 2017, at an English National Health Service (NHS) hospital using the International Classification of Diseases, 10th Edition (ICD-10) code ‘C61’ [10]. The NPCA database includes staging data from the English Cancer Registry [11], administrative hospital data from the Hospital Episode Statistics database [12], and treatment data from the Radiotherapy Dataset (RTDS) [13]. English Cancer Registry data includes all men newly diagnosed with prostate cancer [14]. We excluded 20,538 men with stage M1 or PSA ≥ 100 (17.3%), 4447 men with stage N1 (3.8%), and 31,642 men because of missing staging (26.7%). As a result, 61,999 patients were included in our study.

We classified the included patients according to the CPG classification using Gleason score, PSA level, and TNM as outlined in Table 1. For comparison, we also classified the men according to the three-tiered NPCA risk classification [9], which is based on the NICE recommendation (low, intermediate, and high risk) with modifications to accommodate the unavailability of staging data required to subdivide stage T2 into T2a, T2b, or T2c [1].

Patient characteristics including age, ethnicity (white and non-white), and socioeconomic deprivation status were identified using Hospital Episode Statistics (HES) data. Socioeconomic deprivation status was determined for patients from the English 2015 Index of Multiple Deprivation (IMD) based on their area of residence and divided according to quintiles of the national distribution [15]. We used ethnicity available from the English Cancer Registry to supplement any missing values in HES. The Royal College of Surgeons (RCS) Charlson score was used to identify any comorbid conditions according to diagnosis codes in the HES records within 1 year of diagnosis [16].

### Study outcome

We identified the treatments that men received using HES and RTDS data. The OPCS Classification of Interventions and Procedures (OPCS-4) code ‘M61’ in HES was used to identify men who underwent a primary radical prostatectomy and the date of the operation [17]. The data item ‘treatment modality’ in the RTDS was used to select men who underwent primary radical radiotherapy and the start date of this treatment. Primary radical treatment was defined as either radical radiotherapy or a radical prostatectomy within 1 year of diagnosis. For those undergoing more than one radical treatment, the primary treatment was selected based on the earliest treatment date, i.e. a patient who received surgery followed by radiotherapy was analysed in the surgery group.

### Statistical analysis

Multivariable multilevel Poisson regression with robust standard errors was used to estimate the risk ratio (RR) comparing the proportion of men who had radical treatment, compared to the proportion of men who did not, according to CPG and adjusted for age, Charlson score, socioeconomic deprivation status, and ethnicity [18]. A random intercept was modelled for each hospital to adjust for clustering within hospitals. A further Poisson regression model was used for the men who received radical treatment to estimate adjusted risk ratios comparing the proportion of men having had radiotherapy as their primary treatment, compared to the proportion of men having surgery, according to CPG. Missing data for ethnicity (3.1%) were imputed with multiple imputation using chained equations to create ten data sets. Rubin’s rules were then used to combine the risk ratios across these ten data sets. Wald tests were used to calculate *P* values with significance set at *P* < 0.05.

Between-hospital variation in adjusted radical treatment rates was explored visually using funnel plots to establish whether this variation in the proportion of patients receiving radical treatment was greater than expected by chance alone for each CPG [19]. Control limits corresponding to two standard deviations from the national average population (95%) were set for each CPG funnel plot. The proportion of hospitals outside of these limits was used to quantify the between-hospital variation with a larger proportion indicating greater between-hospital variation.

## Results

### Cohort description

**Fig. 1:**
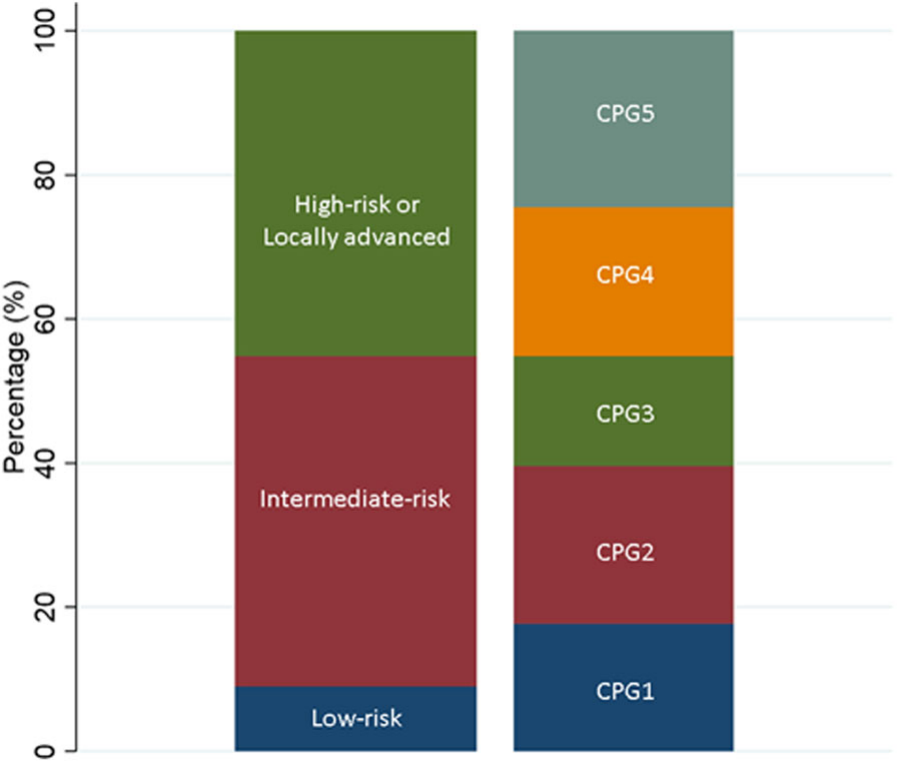
Distribution of risk groups according to the five-tiered Cambridge Prognostic Group (CPG) and the three-tiered National Prostate Cancer Audit (NPCA)/NICE stratification system

Of the 61,999 men with non-metastatic prostate cancer included in the study, 10,963 were grouped in CPG1 (17.7%) as the lowest risk group, 13,588 in CPG2 (21.9%), 9452 in CPG3 (15.2%), 12,831 in CPG4 (20.7%), and 15,165 in CPG5 (24.5%) as the highest risk group (Fig. 1). CPG1 included more men than the 5588 men in the low-risk group of the traditional three-tiered classification given that T2 cases were also included. CPG2 and CPG3 included all remaining men in the intermediate-risk group, and CPG4 and GP5 included all men in the high-risk group.

Apart from the patients’ age, patient characteristics were similar across CPGs (comorbidities, deprivation status, and ethnicity). The proportion of older men (≥ 70 years) increased with CPG (CPG1, 30.7; CPG2, 37.2; CPG3, 52.3; CPG4, 47.8; CPG5, 60.4) (Table 2).

**Table 2:** Patient characteristics of men diagnosed with non-metastatic prostate cancer stratified by the Cambridge Prognostic Group (CPG)

	CPG1		CPG2		CPG3		CPG4		CPG5		All men	
	No.	%	No.	%	No.	%	No.	%	No.	%	No.	%
Age group (years)												
< 60	2637	24.1	2627	19.3	1052	11.1	1553	12.1	1118	7.4	8987	14.5
60–69	4962	45.3	5902	43.4	3455	36.6	5142	40.1	4884	32.2	24,345	39.3
70–79	3033	27.7	4529	33.3	4276	45.2	5315	41.4	6828	45.0	23,981	38.7
≥ 80	331	3.0	530	3.9	669	7.1	821	6.4	2335	15.4	4686	7.6
Number of comorbidities (RCS Charlson score)												
0	8690	79.3	10,672	78.5	7216	76.3	10,009	78.0	11,383	75.1	47,970	77.4
1	1565	14.3	1985	14.6	1531	16.2	1953	15.2	2427	16.0	9461	15.3
≥ 2	708	6.5	931	6.9	705	7.5	869	6.8	1355	8.9	4568	7.4
Deprivation status (national quintiles)												
1 (least deprived)	2720	24.8	3338	24.6	2243	23.7	3048	23.8	3482	23.0	14,831	23.9
2	2620	23.9	3093	22.8	2218	23.5	3028	23.6	3613	23.8	14,572	23.5
3	2249	20.5	2847	21.0	1986	21.0	2715	21.2	3231	21.3	13,028	21.0
4	1881	17.2	2378	17.5	1647	17.4	2198	17.1	2564	16.9	10,668	17.2
5 (most deprived)	1493	13.6	1932	14.2	1358	14.4	1842	14.4	2275	15.0	8900	14.4
Ethnicity												
White	9441	92.7	11,887	92.5	8198	91.6	11,268	93.0	13,437	94.0	54,231	92.9
Non-white	741	7.3	967	7.5	749	8.4	849	7.0	862	6.0	4168	7.1
Missing	781		734		505		714		866		3600	

### Radical treatment by prognostic group

The proportion of men who received radical treatment increased in men with higher risk according to their CPG (*P* < 0.001) and older age (*P* < 0.001), and decreased in men with more comorbidities (Table 3). As expected, men in CPG1 were least likely to receive radical treatment (11.3%). Of the 5588 men in CPG1 with T1 disease, who have low-risk disease according to the three-tiered classification, 5.1% received radical treatment compared to 17.7% of the 5375 men in CPG1 with T2 disease, who have intermediate-risk disease according to the three-tiered system (adjusted RR 3.53; 95% CI 2.61–4.78; *P* < 0.001).

**Table 3:** Radical treatment within 1 year of being diagnosed with prostate cancer according to the Cambridge Prognostic Group (CPG)

	Patients (*n*)	Radical Tx (%)	Adj. RR	95% CI	*P*
CPG					
1	10,963	1236 (11.3)	0.23	0.18–0.30	**< 0.001**
2	13,587	6580 (48.4)	1		
3	9449	6263 (66.3)	1.44	1.36–1.53	
4	12,828	10,112 (78.8)	1.69	1.32–2.15	
5	15,160	11,118 (73.3)	1.70	1.37–2.11	
Age group (years)					
< 60	8987	5195 (57.8)	1		**< 0.001**
60–69	24,343	14,843 (61.0)	0.91	0.82–1.01	
70–79	23,972	14,073 (58.7)	0.78	0.70–0.88	
≥ 80	4685	1198 (25.6)	0.31	0.22–0.44	
Number of comorbidities (RCS Charlson score)					
0	47,960	27,921 (58.2)	1		**0.011**
1	9460	5159 (54.5)	0.95	0.73–1.24	
≥ 2	4567	2229 (48.8)	0.85	0.51–1.44	
Socioeconomic deprivation status (5th of national distribution)					
1 (least deprived)	14,825	8525 (57.5)	1		0.267
2	14,571	8309 (57.0)	0.98	0.92–1.04	
3	13,027	7428 (57.0)	0.98	0.88–1.08	
4	10,666	6067 (56.9)	0.97	0.49–1.76	
5 (most deprived)	8898	4980 (56.0)	0.93	0.60–1.60	
Ethnicity					
White	57,581	32,838 (57.0)	1		0.908
Non-white	4406	2471 (56.0)	0.98	0.87–1.13	

Men in CPG3 were more likely to receive radical treatment than those in CPG2 (66.3% versus 48.4%; adjusted RR 1.44; 95% CI 1.36–1.53; *P* < 0.001). The overall proportion of men receiving radical treatment was slightly higher in men in CPG4 (78.8%) than in CPG5 (73.3%), but this difference was not statistically significant (adjusted RR 1.01; 95% CI 0.98–1.04; *P* = 0.638).

**Fig. 2:**
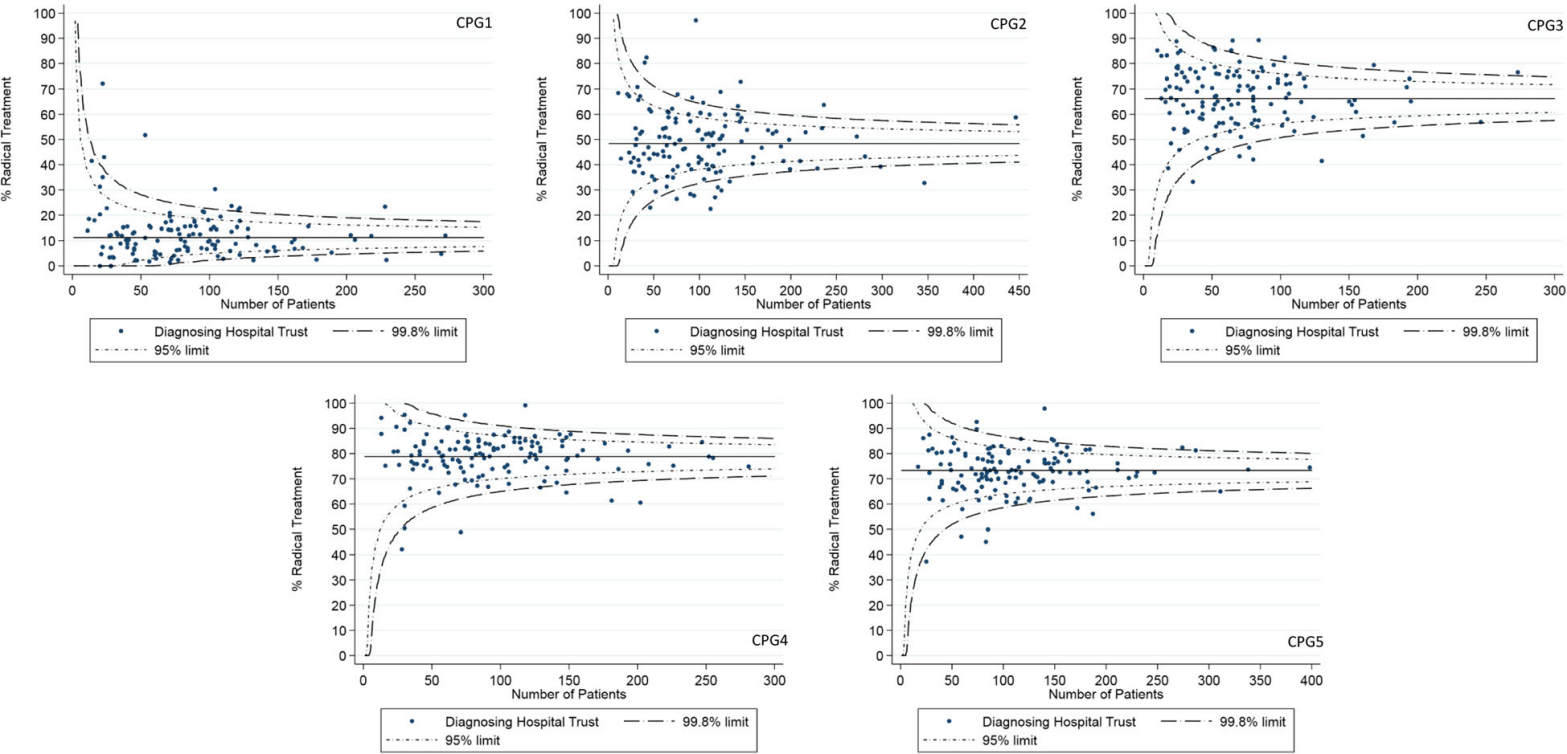
Adjusted funnel plots for the proportion of men undergoing radical treatment within 1 year of diagnosis, according to the Cambridge Prognostic Groups at diagnosis. Each data point represents a hospital where men were diagnosed

Between-hospital variation in radical treatment rates is shown visually for each CPG using funnel plots in Fig. 2. The observed range between the lowest and the highest treatment rates across the 129 hospitals was largest for CPG2 (CPG1, 0 to 72.2%; CPG2, 22.5 to 97.2%; CPG3, 33.2 to 89.3%; CPG4, 42.2 to 99.2%; CPG5, 37.3 to 97.9%). Assuming differences arise from random errors alone, the expected number of hospitals outside the inner (95%) funnel limits for all analyses is 6. 42 hospitals (32.6%) lay outside the inner funnel limits for CPG2, which was comparably higher than for the other CPGs (CPG1, 31 hospitals (24.0%); CPG3, 33 hospitals (25.6%); CPG4, 29 hospitals (22.4%); CPG5, 34 hospitals (26.4%)).

### Treatment allocation by prognostic group

In men who received radical treatment, considerable differences were seen in primary treatment modality according to CPG (Table 4). The proportion of men who received primary radiotherapy according to CPG was similar for men in CPG3 and CPG4: 59.3% of men in CPG3 and 59.1% in CPG4 (adjusted RR 0.99; 95% CI 0.92–1.08). More men in CPG5 however had primary radiotherapy (79.9%) than those in CPG4 (59.1%; adjusted RR 1.26; 95% CI 1.12–1.40). The proportion of men in CPG3 receiving primary radiotherapy (59.3%) was also higher than in CPG2 (43.9%; adjusted RR 1.18; 95% CI 1.10–1.26).

**Table 4:** Mode of treatment (surgery or radiotherapy) for those who underwent radical treatment according to the Cambridge Prognostic Group (CPG)

	Patients (*n*)	Surgery (%)	RT (%)	Adj. RR	95% CI	*P*
CPG						
1	1236	726 (58.7)	510 (41.3)	0.87	0.59–1.28	**< 0.001**
2	6580	3691 (56.1)	2889 (43.9)	0.84	0.80–0.88	
3	6263	2488 (40.7)	3775 (59 .3)	0.99	0.92–1.08	
4	10,112	4133 (40.9)	5979 (59.1)	1		
5	11,118	2238 (20.1)	8880 (79.9)	1.26	1.12–1.40	
Age group (years)						
< 60	5195	3916 (75.4)	1279 (24.6)	1		**< 0.001**
60–69	14,843	7275 (49.0)	7568 (51.0)	1.97	1.68–2.30	
70–79	14,073	2081 (14.8)	11,992 (85.2)	3.17	2.54–3.95	
≥ 80	1198	40 (0.3)	1194 (99.7)	3.52	2.84–4.37	
Number of comorbidities (RCS Charlson score)						
0	27,921	11,120 (39.8)	16,801 (60.2)	1		0.081
1	5159	1577 (30.6)	3582 (69.4)	1.09	1.01–1.18	
≥ 2	2229	579 (26.0)	1650 (74.0)	1.12	0.99–1.27	
Socioeconomic deprivation status (5th of national distribution)						
1 (least deprived)	8525	3387 (39.7)	5138 (60.3)	1		**0.003**
2	8309	3068 (36.9)	5241 (63.1)	1.05	0.87–1.26	
3	7428	2771 (37.3)	4657 (62.7)	1.07	0.77–1.50	
4	6067	2244 (37.0)	3823 (63.0)	1.12	0.74–1.67	
5 (most deprived)	4980	1806 (36.3)	3174 (63.7)	1.16	0.72–1.88	
Ethnicity						
White	32,840	12,078 (36.8)	20,760 (63.2)	1		0.922
Non-white	2469	1198 (48.5)	1273 (51.5)	0.94	0.29–3.08	

In men who received radical treatment, the proportion of men who received primary radiotherapy increased with age (*P* < 0.001), the presence of comorbidities (*P* = 0.081), and higher levels of socioeconomic deprivation (*P* < 0.003; Table 4). For example, 24.6% of men younger than 60 years received primary radiotherapy compared to 99.7% of men aged 80 years and above. Corresponding percentages were 60.2% in men without comorbidities, compared to 74% in men with two or more comorbidities, and 60.3% in men with the lowest level of socioeconomic deprivation, compared to 63.7% in men with the highest level of socioeconomic deprivation.

## Discussion

This study has demonstrated that the five-tiered CPG classification distributes men in groups that are about equal in size. The subdivision of men with intermediate-risk disease into CPG2 and CPG3, and of men with high-risk disease into CGP4 and CPG5, gives a finer and more clinically relevant degree of granularity compared to the traditional three-tiered risk stratification system. It is therefore more informative for studies, such as the National Prostate Cancer Audit, that aim to evaluate the appropriateness of treatment selection amongst providers of prostate cancer services at the regional or national level.

By using the CPG classification, we also demonstrated the considerable between-hospital variation in radical treatment rates observed for men in CPG2 across England, which indicates a lack of consensus in the management of men with ‘favourable’ intermediate-risk disease (i.e. those in CPG2). A potential explanation for this discrepancy may be due to the differences in the uptake of magnetic resonance imaging techniques and image-guided biopsies. However, we could not explore this further due to a lack of imaging data within the data sources that were available.

The recently updated NICE guidelines, published in 2019, currently advise active surveillance for men with intermediate-risk disease only if they opt not to have radical treatment. It is important to note that the NICE guidelines do not make a distinction between favourable (CPG2) and unfavourable (CPG3) intermediate-risk disease. Without this distinction, a clinical emphasis is placed on treatment and not on surveillance for men in CPG2 [1]. Equally, although the European Association of Urology guidelines, published in 2017, make reference to the subdivision of intermediate-risk disease into those with favourable or unfavourable disease, the inclusion criteria for active surveillance do not include patients with favourable intermediate-risk disease [20].

However, other guidelines, such as those supported by Prostate Cancer UK, do indicate that active surveillance is suitable for men who have favourable intermediate-risk disease and a PSA density of ≤ 0.2 ng/ml^2^ [21]. North American guidelines also recommend active surveillance as a primary management option for men with favourable intermediate-risk disease. For example, the American Urological Association recommend offering active surveillance to ‘select’ patients but adds the caveat that patients should be informed that this comes with an increased risk of developing metastases without defining by how much [7]. Also, the National Comprehensive Cancer Network recommends active surveillance specifically for men with favourable intermediate-risk disease but adds that less than 50% of biopsy cores should be positive [8].

Using the CPG classification also sheds light on whether men who received radical treatment had surgery or radiotherapy. Radiotherapy appears to be a more frequent treatment for men with more high-risk disease. Eighty per cent of men in CPG5 underwent radiotherapy compared to 59% in CPG4. Men in CPG5 represent those with more locally advanced disease, for whom surgery may not be considered appropriate. The CPG classification reveals the differences in how men with ‘high-risk’ disease are apparently selected for therapy when subdivided into CPG4 and CPG5, which studies that used the traditional three-tiered classification fail to demonstrate [22].

An important observation from our study is the relationship between age and CPG at diagnosis. The proportion of men diagnosed with CPG5 aged 70 or older was double that of those diagnosed with CPG1, indicating a clear progression of prostate cancer stage with age and that age at diagnosis is a major risk factor for aggressive disease. This observation highlights the complexity of the association of age and disease aggressiveness on the one hand and treatment selection on the other [23, 24], given the clinical imperative to avoid early surgical complications in older patients and to reduce the need for later salvage treatments in patients with higher risk disease [25].

We also found that by using the CPG classification, the rates of surgery exceed radiotherapy only in men with CPG1 and CPG2 disease. Interestingly, in a prior publication, we showed an association between being diagnosed at a hospital with surgical services onsite and being more likely to receive surgery for their prostate cancer, than if these services were not available onsite [24].

A key strength of our study is that we used a contemporary national English cohort, ensuring that treatment patterns were representative of the current nationwide practice. Inclusion of more than 60,000 men who could be classified into a CPG enabled a reliable assessment of treatment patterns.

A major limitation of our work is that we were reliant on the accuracy of the clinical coding in the routinely collected hospital data. However, the accuracy of these data has been shown to be high when compared to clinical notes and is sufficiently robust to support its use in research [26]. It must also be noted that the sub-classification of T stage (i.e. T2a, T2b, and T2c) was not available. However, it is unlikely that the use of this further sub-classification would be beneficial given that this level of staging is known to be frequently inaccurate [27, 28].

A further weakness is that we were unable to classify 27% into a CPG due to missing staging data. This demonstrates a trade-off between a better classification system and a higher level of data completeness when using the five-tiered CPG instead of the three-tiered system. Moreover, varying levels of missing data could also introduce bias. For example, the proportion of men who could not be classified into a CPG varied between hospitals. For hospitals that were identified as outliers with higher than expected radical treatment rates for men in CPG2, we could not classify 21.4% into a CPG group, whereas the corresponding figure was 18.7% for hospitals identified as outliers with a lower than expected radical treatment rate.

## Conclusion

In conclusion, by using the CPG classification, we could demonstrate the potential overtreatment of favourable intermediate-risk disease (CPG2) in England given that emerging evidence suggests that active surveillance is an appropriate management strategy for this patient group. Also, we found substantial differences in how men with high-risk disease (CPG4 and CPG5) are managed, especially with respect to whether they get radiotherapy or surgery. Taken together, these results strongly support the use of a classification of prostate cancer risk that provides a finer degree of granularity than the traditional three-tiered classification.

## Data Availability

The cancer registry data used for this study are based on the information collected and quality assured by the Public Health England’s National Cancer Registration Service (www.ncras.nhs.uk). Access to the data was facilitated by the Public Health England’s Office for Data Release. Hospital Episode Statistics were made available by the NHS Digital (www.digital.nhs.uk; all rights reserved). MGP had full access to all the data in the study and takes responsibility for the integrity of the data and accuracy of the data analysis. Data are not available to other researchers as it uses a registry database of patients providing routinely collected data.
